# Appendiceal endosalpingiosis: a case report of a rare finding from appendicectomy

**DOI:** 10.1093/jscr/rjac402

**Published:** 2022-08-31

**Authors:** Agnish Nayak, Josh Karpes, Kathryn Stewart

**Affiliations:** Department of General Surgery, Campbelltown Hospital, Sydney, Australia; Central Clinical School, University of Sydney, Sydney, NSW, Australia; Department of General Surgery, Campbelltown Hospital, Sydney, Australia; St George Hospital Clinical School, University of New South Wales, Kogarah, NSW, Australia; Department of General Surgery, Campbelltown Hospital, Sydney, Australia

## Abstract

Endosalpingiosis is occasionally incidentally found on histopathologic examination of gynecologic or gastrointestinal specimen, most commonly in the ovary, fallopian tube, omentum and uterus. Recently an association between endosalpingiosis and, ovarian and uterine cancer has been described. Here, we describe a rare case of appendiceal endosalpingiosis mimicking appendicitis. Further prospective studies are required to elucidate the clinical significance of appendiceal endosalpingiosis, the potential association with gynecologic malignancy and implications for management.

## INTRODUCTION

Appendiceal endosalpingiosis is a reportedly rare entity with only few case reports in the literature [1–5]. Endosalpingiosis elsewhere including the ovary, fallopian tube, omentum and uterus has recently been associated with ovarian, fallopian tube, primary peritoneal and uterine cancers [6–8]. In the following case, we describe a presentation of appendiceal endosalpingiosis mimicking appendicitis, discuss the potential clinical significance and explore implications for management.

## CASE REPORT

**Figure 1:**
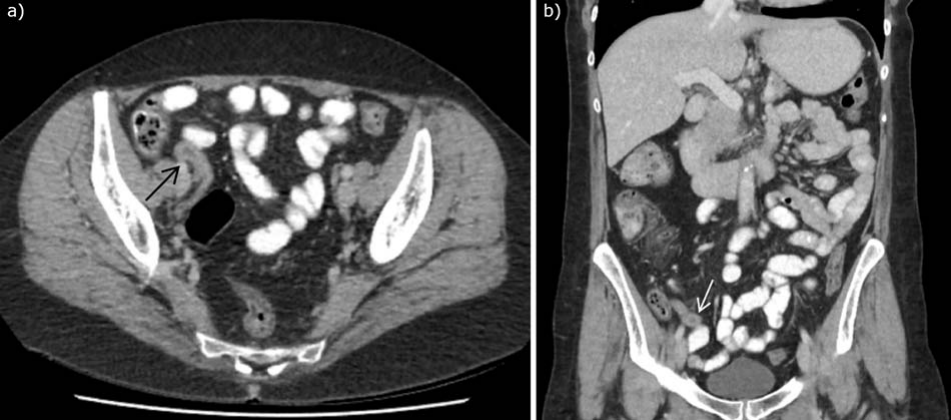
CT abdomen with intravenous and oral contrast demonstrating enlarged appendix, found to be appendiceal endosalpingiosis on histopathology of appendicectomy specimen, in (**a**) axial section and (**b**) coronal section. Arrows to enlarged appendix.

**Figure 2:**
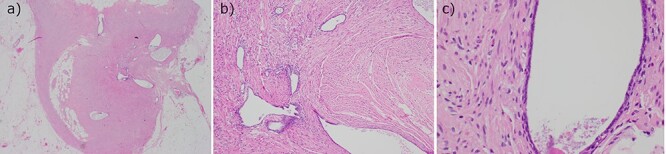
Histopathology of the appendix with hematoxylin and eosin stain demonstrating occasional endometrial type glands with no stroma or hemosiderin macrophages, in keeping with endosalpingiosis. Viewed in (**a**) low, (**b**) intermediate and (**c**) high magnification.

A 56 year old woman presented to emergency with seven days of abdominal pain. Her pain was initially dull and within the peri-umbilical region before migrating to the right iliac fossa and becoming sharp and constant. The pain was associated with subjective fevers and anorexia. There were no bowel or urinary symptoms reported. The patient was post-menopausal and before presentation was previously well with no significant past medical history; her only surgical history included two lower segment caesarean sections. She was seen by her general practitioner who arranged for an outpatient CT of the abdomen and pelvis with oral and intravenous contrast, undertaken on day six of symptoms. CT findings demonstrated a thickened appendix measuring up to 11 mm, reported as consistent with acute appendicitis. There was no significant fat stranding, intraperitoneal free fluid, nor gas, and the CT was otherwise unremarkable (Fig. 1). Upon presentation to emergency, examination found a clinically stable patient with tenderness in the right iliac fossa and suprapubic regions, particularly over McBurney’s point. Rosving’s sign was negative. White cell count was 6.8 × 10^9^ cells/L, neutrophils 2.5 × 10^9 cells/L, C-reactive protein of 9.5 mg/L, while creatinine was slightly elevated at 96 μmol/L and electrolytes were within normal limits. She was incidentally found to be COVID-19 positive, however, was asymptomatic and was fully vaccinated. As her abdominal symptoms continued, she was taken to the operating theatre for a laparoscopic appendicectomy. This was completed unremarkably, with intra-operative macroscopic appearance of a thickened appendix without other abnormality, and the specimen routinely sent for histopathology. She was subsequently discharged from hospital without complication with resolution of her pain. Histopathology demonstrated mild acute mucosal appendicitis with occasional endometrial type glands with no stroma nor hemosiderin-laden macrophages, in keeping with endosalpingiosis (Fig. 2). There was no evidence of malignancy.

## DISCUSSION

Endosalpingiosis is typically considered the presence of ectopic benign glands lined by tubal-type epithelium in the peritoneum or subperitoneal tissues [9]. It is most commonly discovered as an incidental finding upon histopathological examination of tissue from gastrointestinal or gynecologic surgery. Occasionally gross changes can be identified intra-operatively as multiple, punctate fluid-filled small cysts [7]. Reported prevalence of endosalpingiosis within the literature varies from 1 to 22% [6, 8, 10] from pathological analyses of varying populations of patients undergoing gynecologic surgery. Endosalpingiosis is histopathologically distinct from endometriosis due to the ciliated glandular epithelium, lack of endometrial stromal component and lack of an inflammatory response, although endometriosis is reported as present concurrently in roughly one third of cases [6–8].

Endosalpingiosis of the appendix is much rarer, with only five case reports within the literature [1–5]. A large database review of 58 000 procedures from 1998 to 2013 by Esselen et al. found appendiceal endosalpingiosis in only 20 cases. Tudor et al. [1] described two cases similar to that presented here, with patients with right lower quadrant pain but white blood cell count within normal limits. Notably, similar to the cases reportedly by Tudor et al., no significant inflammatory change was identified around the appendix on CT imaging. Endosalpingiosis elsewhere within the gastrointestinal tract is also rare, with scattered case reports identifying its presence within the terminal ileum and rectosigmoid colon [11–13].

Endosalpingiosis has been associated with ovarian and uterine cancer. Hermens et al. [2] reviewed 2500 cases of women with histologically proven endosalpingiosis from 1990 to 2015 and found an age-adjusted incidence rate ratio of 44 (95% CI 35-54). They found this association was strongest with clear cell and endometroid ovarian cancer subtypes, and was independent of concurrent diagnosis of endometriosis. Upon subgroup analysis, they found this association to be predominantly due to cases of synchronous diagnoses of endosalpingiosis and ovarian cancer, specifically diagnoses of one within six months of the other. In their large review, Esselen et al. [3] found a significant association between endosapingiosis and ovarian, fallopian tube, primary peritoneal and uterine cancers. They found this association strongest with the serous borderline subtype of ovarian cancer. Sunde et al. [4] examined a smaller series finding a much higher rate of endosalpingiosis based on an alternative protocol for pathologic assessment, which diminished any association with gynecologic malignancy. Given the relative rarity of endosalpingiosis, recommendations regarding surveillance for malignancy following diagnosis of endosalpingiosis have yet to discussed in the literature.

There is scant literature regarding a potential association between appendiceal endosalpingiosis specifically and gynecologic malignancy. There are no reported rates of endosalpingiosis in series of patients undergoing appendicectomy for suspected appendicitis, as in the currently reported case. Esselen et al. did not present subgroup analysis of the association between their 20 cases of appendiceal endosalpingiosis and ovarian or uterine cancer. The association between endosalpingiosis and gynecologic malignancy may be clinically relevant to endosalpingiosis in acute appendicitis; however, future research is required to further explore this.

## CONCLUSION

In summary, we present a rare case of appendiceal endosalpingiosis as a mimic for appendicitis. Similar to the few reported cases in the literature, pain improved upon appendicectomy. There is limited data to extend the reported association between endosalpingiosis and gynecologic malignancy to appendiceal endosalpingiosis. It is therefore unclear whether patients with incidental appendiceal endosalpingiosis should be offered further investigation or closer monitoring for earlier detection of possible uterine or ovarian cancers. Further studies are required to more closely examine the relationship between appendiceal endosalpingiosis and gynecologic malignancies in order to assist surgeons who encounter this potentially under-reported mimic for appendicitis.

## CONFLICT OF INTEREST STATEMENT

The authors declare that there is no conflict of interest regarding the publication of this paper.

## FUNDING

None.
